# Circadian rest-activity rhythm disorders in advanced cancer: assessment, diagnosis and clinical correlates

**DOI:** 10.1136/spcare-2025-005410

**Published:** 2025-10-02

**Authors:** Craig Gouldthorpe, Andrew Neil Davies

**Affiliations:** 1 School of Medicine, Trinity College Dublin, Dublin, Ireland; 2 Academic Department of Palliative Medicine, Our Lady’s Hospice & Care Services, Dublin, Ireland

**Keywords:** Cancer, Clinical assessment, Palliative Care, Quality of life, Symptoms and symptom management, Supportive care

## Abstract

**Introduction and aims:**

Circadian rest-activity rhythms describe patterns in rest and physical activity across and between 24-hour periods. Research highlights important associations between circadian disruption, including the rest-activity rhythm, and clinical outcomes in patients with cancer. This study aimed to assess the circadian rest-activity rhythms, and prevalence of circadian rest-activity rhythm disorders (CARDs), in patients with advanced cancer.

**Methods:**

An observational study of 72 outpatients with locally advanced or metastatic cancer took place over a 1-year period, considering objective (accelerometry) and subjective (patient diary) measures of circadian rest-activity rhythms, patient-reported outcomes (Memorial Symptom Assessment Scale-Short Form, European Organisation for Research and Treatment of Cancer Core Quality of Life Questionnaire, brief Pittsburgh Sleep Quality Index and Epworth Sleepiness Scale) and clinical markers.

**Results:**

CARDs affected up to 60% of patients with advanced cancer. Increased circadian disruption was seen in patients with more advanced disease, particularly with metastatic organ involvement (p=0.038), poorer performance status (p=0.018), higher inflammatory status (p=0.018), anaemia (p=0.007) and iron deficiency (p=0.002). The study also highlights that patients with advanced cancer and a CARD diagnosis have a higher symptom burden, particularly fatigue (p=0.003) and drowsiness (p=0.005), higher symptom-related distress (p<0.001), a poorer Global Health tatus (p=0.005) and poorer functioning subscales (p<0.014).

**Conclusion:**

This is the first study to assess circadian rest-activity rhythms in accordance with new assessment and diagnostic guidelines. Further research is now required to validate the diagnostic criteria, standardise technical approaches to assessment and consider risk factors for the development of a CARD and additional clinical outcomes of interest.

**WHAT IS ALREADY KNOWN ON THIS TOPIC**

- Circadian rhythms are commonly disordered in patients with advanced cancer. Disordered circadian rhythms may impact clinical outcomes, including worse symptomatology, quality of life and survival. Approaches to investigating and reporting circadian rhythms have previously lacked standardisation. However, recent guidelines outline an approach to the assessment of an observable circadian rest-activity rhythm and the diagnosis of circadian rest-activity rhythm disorders (CARDs) in patients with cancer. This study assessed the circadian rest-activity rhythms, prevalence of CARDs and associations with patient characteristics and clinical outcomes in a cohort of patients with advanced cancer.

**WHAT THIS STUDY ADDS**

- CARDs are common, affecting up to 60% of patients with advanced cancer. Circadian rest-activity rhythms are commonly disordered due to daytime sedentariness and night-time restlessness, but also include variation in rest and physical activity rhythms between days. Potential risk factors for a CARD include more advanced disease, particularly metastatic organ involvement, poorer performance status, higher inflammatory status, anaemia and iron deficiency. Improved circadian rest-activity rhythms were seen in those who share a bed. A diagnosis of a CARD, in patients with advanced cancer, is associated with increased symptom burden, particularly fatigue and drowsiness, and symptom-related distress. Those with a CARD diagnosis also had poorer Global Health Status and functioning subscales.

**HOW THIS STUDY MIGHT AFFECT RESEARCH, PRACTICE OR POLICY**

- The findings of this study warrant further investigation of CARDs in patients with cancer. Research needs to address similarities and differences between different cancer types and further elucidate other risk factors for the development of the diagnosis. More work is also required to examine the implications of the diagnosis on additional clinical outcomes, including the risk of clinical deterioration, hospital admissions and overall prognosis.

## Introduction

 Circadian rhythms describe physiological and biochemical changes that occur across 24-hour periods.^1^ Such changes may be demonstrated in observable behaviours, such as sleeping, eating and physical activity, but also in measurable serological changes, such as levels of cortisol and melatonin. Cells throughout the human body function in a circadian fashion under the instruction of several ‘clock’ genes, predominantly period (per), cryptochrome (cry), circadian locomotor output cycles kaput (CLOCK) and Brain and Muscle ARNT-Like 1 (BMAL1) genes.^1^ However, for physiological processes to be effective, tissues and organs need to function synchronously, and the peripheral clock machinery is coordinated by a central pacemaker, the suprachiasmatic nuclei (SCN), within the hypothalamus.^1^ The SCN can also interpret environmental factors, predominantly the presence or absence of light, in coordinating internal processes with our environment.^1^

Circadian disruption is common among patients with cancer and appears to be more pronounced with advancing disease.^2^ There are several postulated mechanisms leading to circadian disruption within cancer diagnoses, and the association with circadian disruption appears bidirectional. Thus, the presence of cancer, along with anticancer therapies, disrupts physiological and sleep circadian rhythms. Furthermore, circadian disruption may promote cancer development and, in those with a cancer diagnosis, be related to more rapid disease progression.^3^ Circadian disruption has been linked to important clinical outcomes for patients with cancer, including symptom burden, quality of life measures and survival.^2^

A circadian rhythm of interest is the circadian rest-activity rhythm. This describes patterns of rest (physical inactivity) and physical activity across the 24-hour period and also between 24-hour periods.^4^ Patients with disruption of this circadian rest-activity rhythm may demonstrate daytime sedentariness and night-time restlessness, a significant variation in rest-activity patterns between days or a combination of the two. Potentially reversible modifiers of the circadian rest-activity rhythm should be considered in this patient group and may include poorly managed symptoms, sleep-related disorders, blood and electrolyte abnormalities and certain medications.^4^

Previous studies demonstrate a lack of consistency in methodology (and reporting) of circadian rest-activity rhythms. Recently, however, an international group of experts developed guidelines for the standardised assessment and diagnosis of Circadian rest-Activity Rhythm Disorders (CARDs) in patients with cancer.^4^ This includes the use of a clinical review, patient diary completion and accelerometry monitoring. Accelerometry devices are worn on the body and measure levels of activity across specified time periods (epochs), with 60s epochs being commonly used.^5^ Accelerometry allows for a non-invasive and continuous measure of physical activity levels and has been found to be a valid and acceptable approach for patients with cancer (see methodology).^6^

This is the first study to assess circadian rest-activity rhythms in accordance with new assessment and diagnostic guidelines. The study objectives were:

- Describe circadian rest-activity rhythms in patients with advanced cancer.
- Identify the prevalence of CARDs in patients with advanced cancer according to the newly formed diagnostic criteria.
- Identify important associations with CARDs.
  - Patient characteristics.
  - Symptom and quality of life measures.

## Methods

This prospective, observational study was conducted at a teaching hospital and hospice service in Dublin. The study recruited outpatients with locally advanced or metastatic cancer and was conducted over a 1-year period. Ethical approval was granted by the joint St James’ Hospital–Tallaght University Hospital Research Ethics Committee (ID 1926). The study was registered on ClinicalTrials.gov (NCT06023654).

The inclusion criteria were: (i) outpatient; (ii) ≥18 years old; (iii) diagnosis of advanced cancer (locally advanced, incurable, metastatic); (iv) ambulatory and (v) estimated prognosis ≥3 months. The exclusion criteria were: (i) inpatient; (ii) engaged in shift work; (iii) long haul travel in the last 14 days and (iv) cognitive impairment limiting the ability to complete the assessment tool and/or patient diary. All patients who met these criteria were eligible to take part in the study.

Potential participants were identified by the clinical team and, with permission, were then approached by the researcher. Patients were provided with a patient information leaflet and given the opportunity to ask the researcher questions. Participants attended at the beginning of a week, provided written consent, and then completed baseline assessments, including demographic and lifestyle information, a clinical history and examination, performance status (Eastern Cooperative Oncology Group score), cognition (Abbreviated Mental Test Score), physical and psychological symptoms (Memorial Symptom Assessment Scale-Short Form (MSAS-SF)), quality of life (European Organisation for Research and Treatment of Cancer Core Quality of Life Questionnaire (EORTC-QLQ-C30)) and chronotype (Morningness-Eveningness Questionnaire).

At the same time, a wrist accelerometer (Micro Motionlogger® watch, Ambulatory Monitoring, USA) was applied to the non-dominant wrist, and a thigh accelerometer (ActivPal, PAL technologies, UK) was placed in a nitrile sleeve and applied to the middle and anterior aspect of the right thigh before securing with a Tegaderm^TM^. Instructions were provided on device care and how to use the event recorder on the Micro Motionlogger® watch to capture the time of getting in and out of bed. Furthermore, participants were provided with a novel Sleep and Activity Diary (online supplemental appendix 1), devised following recently formed recommendations for the assessment of circadian rest-activity rhythms, and instructed on how to use it.^4^

After a 72-hour period of monitoring, participants returned to complete further assessments relating to sleep quality (brief Pittsburgh Sleep Quality Index (bPSQI)), daytime sleepiness (Epworth Sleepiness Scale), obstructive sleep apnoea (STOP-Bang) and presence of restless legs syndrome.

The recently formed recommendations and diagnostic criteria (see table 1) highlight the importance of specific circadian parameters, including the dichotomy index (I<O, proportional activity in bed that is below the median level of activity out of bed), the 24-hour autocorrelation coefficient (r24, a correlation of activity levels at the same time point between days) and the sleep efficiency (SE, amount of time in bed spent sleeping). Studies have reported variable abnormal circadian parameter values; however, median values within participant groups have also been reported. For this study, threshold values to determine abnormality included an I<O<97.5%, r24<0.35 and SE<86%. These values are based on previous studies which highlight the threshold values to be below average and associated with poorer clinical outcomes.^2^

**Table 1:** Diagnostic criteria for circadian rest-activity rhythm disorders (CARDs) in patients with cancer^4^

Criteria	Description
1.	Patients exhibit relatively less daytime physical activity and more night-time physical activity; **OR** rest and physical activity spread across the 24-hour period, rather than distinct rest and active periods **OR** a lack of regularity in rest and active periods between days
2.	The altered physical activity pattern has been present for at least 1 month has a clinical impact on the patient is demonstrable by objective and subjective measures
3.	The altered rest-activity rhythm is not primarily due to another cause

Circadian parameters were calculated from the Micro Motionlogger® watch accelerometry data using dedicated software (Action-W 2.7 3401 and Action-4 V.1.16). The software used the zero-crossing mode, which assesses the frequency of movement above a threshold, and considered 1-min epochs (intervals). Several circadian parameters require an accurate timing of when the participant gets in (DOWN) and out of bed (UP). There is no universal agreement on how this should be determined. For this study, participant-reported DOWN and UP times, via the patient diary or Micro Motionlogger® watch event marker, were reviewed. The self-reported DOWN and UP times were then adjusted to the nearest objective transition from upright to supine, and vice versa, as identified by the thigh accelerometry (ActivPal^TM^). Data were reviewed and edited to remove likely off-wear time of the watch, considering a lack of activity, fall in temperature or absent activity in the life measures channel. The data frame was then adjusted to include a 72-hour window, as advised by the recently formulated guidelines to diagnose a CARD, up until the final UP time on the morning of the second assessment. The proposed sample size (100) was pragmatic, given that the prevalence of CARD was unknown in this cohort of patients. The actual sample size (72) reflects the strict timeframe (12 months) for completion.

Descriptive statistics were primarily used to describe the data, with the normality of the data assessed using the Kolmogorov-Smirnov statistic. Mann-Whitney and Spearman’s rho correlation were used to consider correlations and differences between groups for non-normally distributed data and independent t-tests and Pearson’s correlation to consider correlations and differences between groups for normally distributed data. χ^2^ test was also used to consider associations between dichotomous variables.

The study protocol and all participant-facing information were reviewed by the Voices4Care team, which is a patient and public involvement group consisting of people with palliative care needs, carers and volunteers.

## Results

**Figure 1:**
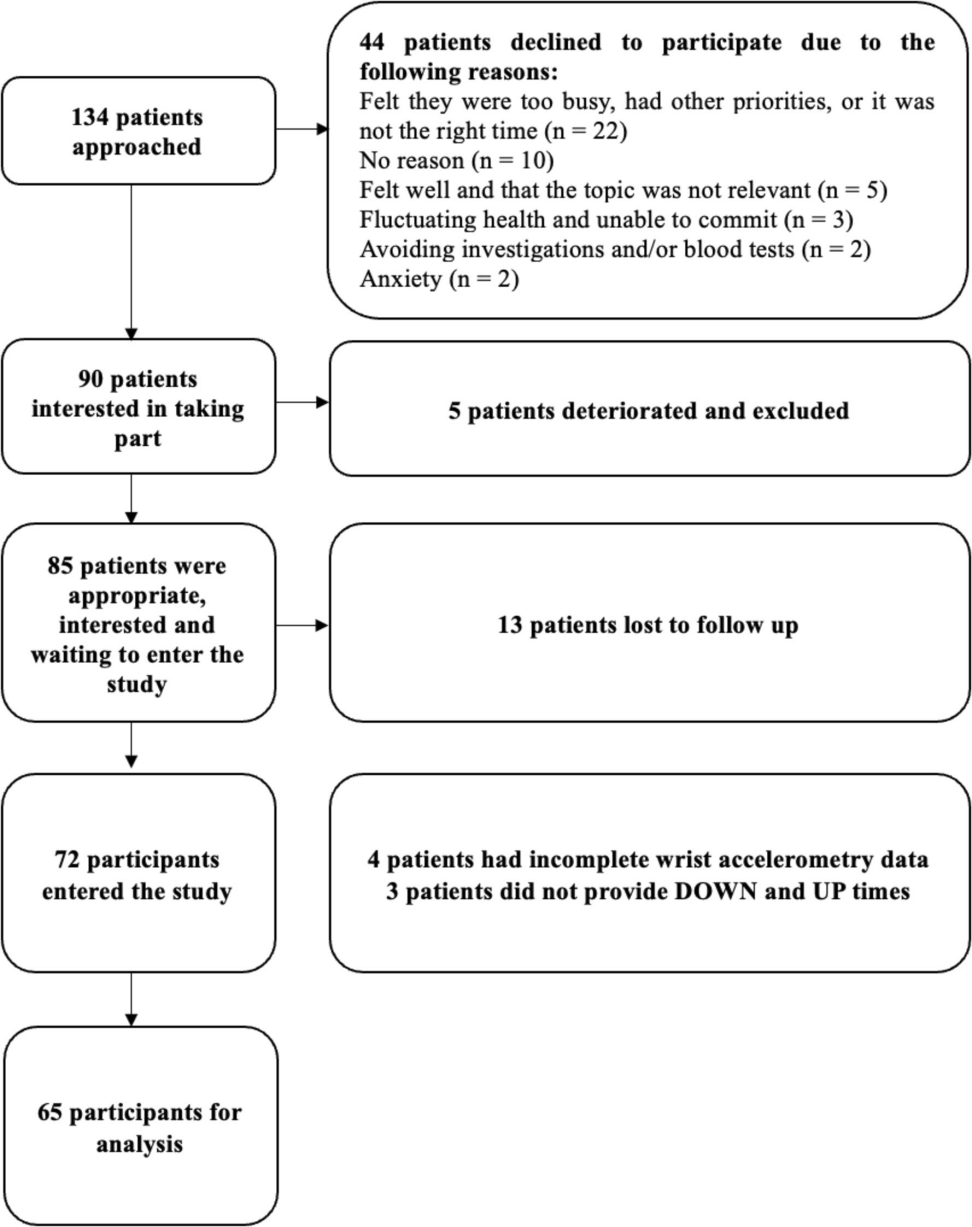
Flow diagram of participants and potential participants in the observational study. The figure demonstrates those approached, interested and entering the study alone with reasons for declining or exiting the recruitment process.

72 patients were recruited over a 12-month period (see figure 1): patients were recruited from oncology hospital outpatients (n=33, 46%), palliative care hospice outpatients (n=20, 28%), community palliative care teams (n=14, 19%) and palliative care hospital outpatients (n=5, 7%). 65 participants had 72 hours of wrist accelerometry data, and self-reported sufficient DOWN and UP times, to allow for relevant data analysis. Four patients had incomplete wrist accelerometry data due to technical issues with the Micro Motionlogger® watch, and three did not have self-reported measures of DOWN and UP times. The baseline characteristics for participants completing the study with sufficient accelerometry and diary data for analysis (n=65) are detailed in online supplemental appendix 2.

### Circadian rest-activity rhythms

**Figure 2:**
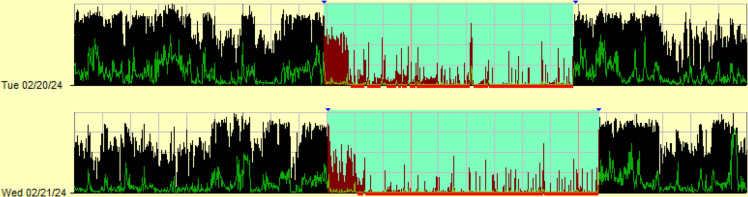
A linear actigram illustrating a ‘good’ dichotomy index (I<O). The black lines represent activity levels per minute. There is a clear distinction between IN-bed (blue zone) and OUT-of-bed activity, with proportionally higher activity levels OUT-of-bed. Blue triangles represent patient-activated event markers. The red line indicates automated software-defined sleep, and the absence of a red line indicates wake.

The median dichotomy index (I<O) for this patient cohort was 95.05% (range 78.51%–99.87%). Participants with ‘good’ and ‘bad’ dichotomy indices are demonstrated in figures2  3. 45 participants (69.2%) had an ‘abnormal’ I<O (below 97.5%). Lower (worse) dichotomy index values were seen in patients with metastatic organ involvement (p=0.038), those no longer receiving anticancer therapy (p=0.08), patients with a higher C-reactive protein (CRP) (>5.0, p=0.018) and those with a Eastern Cooperative Oncology Group (ECOG) score of 2 compared with an ECOG score of 0 (p=0.018). Lower dichotomy index values were seen in patients who did not share a bedroom (p=0.009) and who did not drink alcohol (p=0.037). There was also a significant correlation between the dichotomy index and bilirubin level (r=0.251, p=0.045) and albumin (r=0.274, p=0.028).

**Figure 3:**
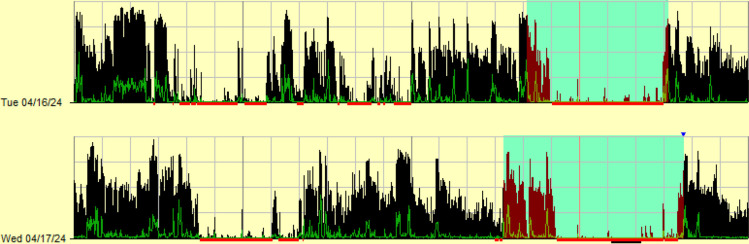
A linear actigram demonstrating a ‘bad’ dichotomy index (I<O). In contrast to figure 2, there is a lack of distinction between rest and activity levels IN (blue zone) and OUT of bed.

In-bed activity was higher in participants with a below average dichotomy index compared with participants with an above average dichotomy index (median=33.07 vs 21.93, U=254, p<0.001). Out-of-bed activity was significantly higher in participants with an above average dichotomy index compared with participants with a below average dichotomy index (median=195.41 vs 134.73, U=902, p<0.001). A combination of higher in-bed activity and lower out-of-bed activity was therefore seen in participants with a worse dichotomy index.

The mean r24 was 0.405 (range 0.13–0.72). 28 participants (43%) had an ‘abnormal’ r24 (<0.35). Participants with a lower (poorer) r24 included those who did not share a bedroom (p=0.041), had a raised CRP (>5, p=0.002) or were smokers (p=0.025). r24 values were correlated with haemoglobin (r=0.34, p=0.007), iron levels (r=0.38, p=0.002), transferrin saturation (r=0.36, p=0.003), sodium (r=0.35, p=0.005), alanine transaminase (ALT) (r=0.271, p=0.03), folate levels (r=0.29, p=0.019), white cell count (WCC) (r=−0.33, p=0.008), CRP (r=−0.36, p=0.004) and alkaline phosphatase (ALP) (r=−0.25, p=0.043).

Median SE was 90.35% (range 51.00%–98.36%), with 51 participants (78%) having an SE <86%. SE was significantly worse in those receiving hormonal and chemotherapy treatments compared with combination anticancer therapies (p=0.018 and p=0.043, respectively). Higher SE was associated with higher haemoglobin levels (p=0.04), higher bilirubin levels (p=0.02) and lower ALT levels (p=0.03).

A large positive correlation was demonstrated between the I<O and r24 (r=0.703, p<0.001) and the I<O and SE (r=0.515, p<0.001). Higher I<O, representing proportionally less in-bed to out-of-bed activity, was associated with more day-to-day regularity in the rest-activity rhythms and more time spent sleeping between sleep onset and sleep offset. A small positive correlation was demonstrated between the r24 and sleep efficiency (r=0.284, p=0.022).

### Diagnosis of a CARD

**Figure 4:**
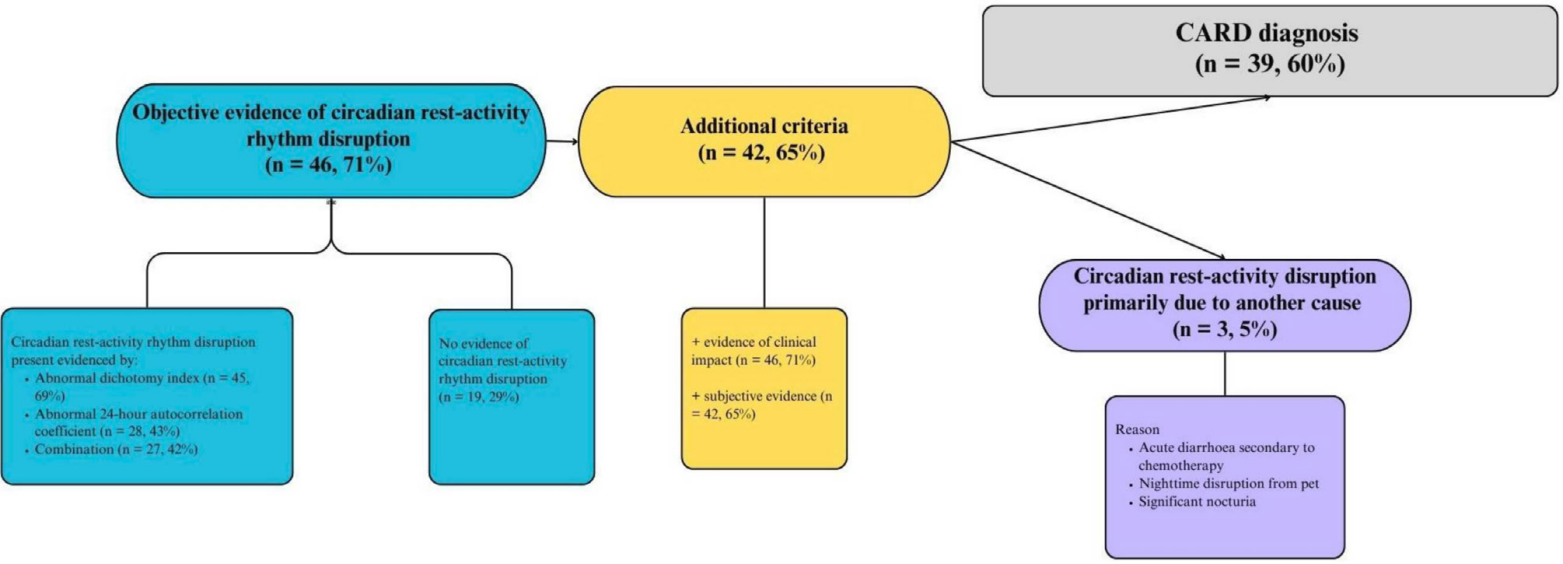
A flow chart demonstrating the process of diagnosing a circadian rest-activity rhythm disorder (CARD).

39 (60%) participants with sufficient data had evidence of a CARD diagnosis (see figure 4).

### Associations with a circadian rest-activity rhythm disruption and a CARD diagnosis

Smokers were more likely to have a CARD diagnosis (p=0.026); however, the diagnosis was not associated with other specific demographic, cancer-related, cancer treatment-related or social factors. A CARD diagnosis was associated with a significantly lower haemoglobin (11.69 g/dL vs 12.78 g/dL, p=0.010), albumin (34.28 g/L vs 36.64 g/L, p=0.019), iron (13.79 umol/L vs 20.96 umol/L, p=0.002) and transferrin saturation levels (24.86% vs 34.87%, p=0.011). Those with a CARD diagnosis also had higher CRP levels (8.8 mg/L vs 3.9 mg/L, p=0.009).

A CARD diagnosis was significantly associated with a greater number of MSAS-identified symptoms (12 vs 9, p=0.018) and the presence of loss of energy (p=0.003), feeling drowsy (p=0.005), shortness of breath (p=0.028), change in taste (p=0.013) and constipation (p=0.028). A CARD diagnosis was also significantly associated with a higher MSAS-SF Global Distress Index (1.31 vs 0.73, p = <0.001), higher Psychological Distress Scores (1.21 vs 0.65, p=0.005), and higher Physical Distress Scores (U=729, p=0.003).

Considering the EORTC-QLQ-C30, those with a CARD diagnosis also had poorer Global Health Status (median 58.33 vs 79.17, p=0.005), poorer physical functioning scores (median 66.67 vs 86.67, p ≤0.001) and poorer role functioning scores (median 59.83 vs 80.13, p=0.014). Participants with a CARD diagnosis also had higher scores for fatigue (median 55.56 v. 22.22, p<0.001), short of breath (33.33 vs 0.00, p=0.005) and constipation (median 33.33 vs 0.00, p=0.026).

Participants with a CARD diagnosis did not have significantly different bPSQI scores (5.62 vs 5.84, p<0.816). Higher ESS scores, representing increased daytime sleepiness, were seen in those with a CARD diagnosis (6.54 (normal daytime sleepiness) vs 3.65 (lower normal daytime sleepiness), p=0.003).

## Discussion

This study describes the circadian rest-activity rhythms within a cohort of patients with advanced cancer and is the first to assess the newly formed diagnostic criteria for a CARD. The study highlights CARDs to be prevalent among this patient group, affecting up to 60%, along with potential risk factors and several important associations with symptomatology and quality of life measures.

The mean I<O within this study (95.05%) resembled, but was lower than, previous reports of 97%–97.5% in patients with cancer; although some studies have focused solely on patients with metastatic colorectal cancer.^7^,^9^ In other words, patients were more active (restless) in bed and less active while out of bed. Although this study reported an average r24 of 0.41, it is important to note that previous reports have highlighted significant variation in group-level circadian parameters, which was seen in this study (r24 (0.13–0.72), I<O (78.51%–99.87%) and SE (51.00%–98.36%)), and with no agreed threshold level, it is difficult to make an adequate comparison between studies.^2^

Subjective sleep disturbance was found in 48%–60% of participants depending on the assessment tool used (MSAS-SF (60%), EORTC-QLQ-C30 (57%) and bPSQI (48%)). This is, as expected, higher than that of patients with cancer generally (40%), as patients with more advanced cancer tend to experience more sleep disturbance, but lower than previous reports of patients with advanced cancer (~70%).^10^,^12^

Previous work has considered potential risk factors for circadian disruption in patients with cancer and has included male sex, more advanced disease, chest wall disease, hormone use, radiotherapy and African-American ethnicity in females.^2^ Similarities were noted within this study, with more significant circadian disruption in those with more advanced disease (particularly metastatic organ involvement) and poorer performance status. Interestingly, sharing a bed was associated with improved circadian rhythmicity as reflected by better day–night distinction and day-to-day regularity. Correlations in sleep duration and 24-hour activity levels between cosleepers have been demonstrated, and interventions to improve circadian rhythmicity should consider the involvement of cohabitants.^13^ A CARD diagnosis was also associated with higher inflammatory status (raised CRP), anaemia and iron deficiency and in those off systemic anticancer therapy, which may be reflective of more advanced disease. Although significant correlations were identified in this study, it is important to highlight that these were all small–medium in size (r<0.038).

Participants with evidence of a CARD had increased symptom burden, particularly fatigue and drowsiness, and higher symptom-related distress. Those with a CARD diagnosis also reported poorer Global Health Status and functioning subscales. These findings are supported by previous reports, whereby symptoms such as pain, sleep disturbance and fatigue were associated with abnormal I<O and r24 values.^2^ This study highlights significant clinical implications for patients with advanced cancer who have a CARD diagnosis and the need for ongoing research in the area, including the consideration of management options to improve circadian rhythmicity and, potentially, clinical outcomes for this patient cohort.

The external validity of the findings is strengthened by including an array of cancer diagnoses. However, small sample sizes for individual cancer groups limit the ability to detect significant intergroup findings. Furthermore, most participants had an ECOG status of 0 or 1, which limits generalising the findings to those with a poorer performance status. Previous studies have highlighted how those with more advanced disease, and thus poorer performance status, have more disordered circadian rhythms than may be evident in the study results.^2^ Furthermore, ethnicity is known to contribute to circadian rest-activity rhythms, and, as 92% of participants were white Irish, the findings of this study cannot be generalised to other ethnic groups.^14^

Circadian rhythms are complex and influenced by several external factors. This study successfully provided a comprehensive assessment of patient-related factors and potential contributing factors. However, not all known confounding factors have been captured, and unknown confounding factors are likely. This includes not capturing diet and feeding patterns, which are known entrainers of several circadian rhythms, including sleep and endocrine function, and should be considered in future research as predictors of the diagnosis.^15^ Although prescribed medication was considered, previous use of recreational drug use, or drug use during the study, was not captured and may, if present, have impacted daytime sleepiness, sleep quality and sleep disruption.^16^ Furthermore, although the participant’s living situation was considered, it did not capture the presence of domestic animals. People with cosleeping arrangements with their pets, for example, report altered sleeping habits, and pet ownership is associated with more frequent daytime physical activity and positive psychological effects, which may influence clinical outcomes of interest.^17 18^ Furthermore, by considering several factors in relation to a CARD diagnosis, there is an inherent risk of false positive results.

There is a lack of agreed standards on the optimum device, data processing and data analysis with regard to accelerometry, circadian rest-activity rhythm assessment and their use in patients with cancer. It remains unclear how to accurately assess their DOWN and UP times, and the study highlights variation between patient-reported and objective measures of these time points. Previous research has highlighted inconsistency in the reporting of sleep parameters, with attribution from a lack of consensus for sleep onset and offset.^19^ A participant’s time in bed may be defined from the time they have physically got into bed, turned off the lights or attempted to fall asleep, and, similarly, time out of bed is defined by the waking up time, the time lights were turned on or the time that they have physically got out of bed.^19^ The differences in circadian parameter values between studies may be expected, given the variable methods of assessing DOWN and UP, the use of different devices and variable data processing and analysis methods. Future research should consider standardising assessment practice further and define the optimum approach for DOWN and UP time detection.

The study assessed participants for a 72-hour window midweek as per the new guidance. Although similar dichotomy index values have been reported when captured midweek or on a weekend, the reporting study did not detail the employment status of participants.^20^ 22% of participants in this study were employed, and further research is required to establish if circadian parameters differ significantly between midweek and weekend days in this group of participants.

Technical issues with the devices, and increased participant engagement, were found more so for wrist accelerometry compared with thigh accelerometry. However, research on circadian rest-activity rhythms in cancer has focused on wrist accelerometry. It remains unclear how the circadian parameter values differ between the two approaches. Threshold values to identify normal and abnormal remain unknown. When considering the diagnostic criteria, it is interesting to note that most participants reported symptom burden and interference with quality of life and/or daily activities, irrespective of whether they had objective or subjective evidence of circadian rest-activity rhythm disruption. The diagnostic criteria also stipulate the altered rest-activity rhythm must be present for at least a month. In this study, participants were asked to consider changes in their daytime and night-time activity over the preceding month as a measure. This is a subjective approach, and devices, such as ActivPal^TM^, can involve minimal participant burden and the option for prolonged monitoring, which allows for an objective alternative to this approach.

## Conclusion

CARDs are common in patients with advanced cancer with important associations with symptomatology, symptom-related distress and quality of life measures. Further research is warranted to assess the risk factors for developing such a disorder and consider additional associations with clinical outcomes of interest. Furthermore, standardisating the technical approach to assessment is required, and consideration of management options to improve circadian rest-activity rhythms, and potentially clinical outcomes, in patients with cancer.

## Supplementary material

10.1136/spcare-2025-005410 online supplemental file 1

10.1136/spcare-2025-005410 online supplemental file 2

## Data Availability

Data are available upon reasonable request.
