# Supplementary material for: Circadian rest-activity rhythm disorders in advanced cancer: assessment, diagnosis and clinical correlates
Source: BMJ Support Palliat Care. 2025 Oct 2;16(1):e005410. doi: 10.1136/spcare-2025-005410 (PMC12772542; doi:10.1136/spcare-2025-005410)
Supplement: online supplemental file 1 [file spcare-16-1-s001.docx]

**Sleep and Activity Diary**

| Participant no: |
| --- |

| **SLEEP AND ACTIVITY DIARY**  (Please complete in the **EVENING**) | **Day 1** | | **Day 2** | | **Day 3** | | **Day 4** | |
| --- | --- | --- | --- | --- | --- | --- | --- | --- |
|  | DD/MM | | DD/MM | | DD/MM | | DD/MM | |
| How sleepy or tired have you felt today? (Please circle) | Extremely sleepy or tired | | Extremely sleepy or tired | | Extremely sleepy or tired | | Extremely sleepy or tired | |
|  | Quite sleepy or tired | | Quite sleepy or tired | | Quite sleepy or tired | | Quite sleepy or tired | |
|  | A little sleepy or tired | | A little sleepy or tired | | A little sleepy or tired | | A little sleepy or tired | |
|  | Not sleepy or tired at all | | Not sleepy or tired at all | | Not sleepy or tired at all | | Not sleepy or tired at all | |
| If you had a nap during the day, how many times did you nap? |  | |  | |  | |  | |
| If you had a nap today, how many minutes did you nap for? | min | | min | | min | | min | |
| If you had a nap today, what time(s) did you nap? (Please circle all that apply) | Morning | | Morning | | Morning | | Morning | |
|  | Afternoon | | Afternoon | | Afternoon | | Afternoon | |
|  | Evening | | Evening | | Evening | | Evening | |
| Did you take any extra medications or drugs today? (Please circle) | Yes | No | Yes | No | Yes | No | Yes | No |
| If you did, what did you take? |  | |  | |  | |  | |
| How many cigarettes did you smoke today? |  | |  | |  | |  | |
| What time did you smoke your last cigarette of the day? | HHMM | | HHMM | | HHMM | | HHMM | |

| Participant no: |
| --- |

| **SLEEP AND ACTIVITY DIARY**  (Please complete in the **EVENING**) | **Day 1** | | **Day 2** | | **Day 3** | | | **Day 4** | | |
| --- | --- | --- | --- | --- | --- | --- | --- | --- | --- | --- |
|  | DDMM | | DDMM | | DD/MM | | | DD/MM | | |
| How many caffeinated drinks did you have today? (e.g. coffee, tea, caffeinated fizzy drinks) |  | |  | |  | | |  | | |
| If you had a caffeinated drink, what time did you have your last caffeinated drink today? | HHMM | | HHMM | | HHMM | | | HHMM | | |
| How many alcoholic drinks did you have today? |  | |  | |  | | |  | | |
| If you had an alcoholic drink, what time did you have your last alcoholic drink? | HHMM | | HHMM | | HHMM | | | HHMM | | |
| Have you spent time doing vigorous physical activity today? (e.g. running, jogging, fast cycling, carrying heavy objects) | Yes | No | Yes | No | Yes | No | | Yes | No | |
| Have you spent time doing moderate physical activity today? (e.g. brisk walking, steady cycling, hoovering, washing windows) | Yes | No | Yes | No | Yes | No | | Yes | No | |
| Have you spent time doing low level physical activity today? (e.g. slow walking, cooking, washing dishes) | Yes | No | Yes | No | Yes | No | | Yes | No | |
| How many hours have you spent lying, sitting, or relaxing today? |  | |  | |  | | |  | | |
| Whilst being active, did you experience any symptoms? (e.g., pain, tiredness, breathlessness) | Yes | No | Yes | No | Yes | | No | Yes | | No |
| If so, what symptoms? |  | |  | |  | | |  | | |

| Participant no: |
| --- |

| **SLEEP AND ACTIVITY DIARY**  (Please complete in the **EVENING**) | | Day 1 | Day 2 | Day 3 | Day 4 |
| --- | --- | --- | --- | --- | --- |
|  |  | DDMM | DDMM | DDMM | DDMM |
| Please highlight the most that you have exerted yourself today on a scale of 0-10 | | | | | |
| 0 | Rest |  |  |  |  |
| 1 | Really easy |  |  |  |  |
| 2 | Easy |  |  |  |  |
| 3 | Moderate |  |  |  |  |
| 4 | Sort of hard |  |  |  |  |
| 5 | Hard |  |  |  |  |
| 6 |  |  |  |  |  |
| 7 | Really hard |  |  |  |  |
| 8 |  |  |  |  |  |
| 9 | Really, really, hard |  |  |  |  |
| 10 | Maximal: just like my hardest race |  |  |  |  |

| What time would you like to be asleep for? (e.g. 10pm – 6am) |  |
| --- | --- |
| What time in the day are you most alert? (e.g. noon – 2pm) |  |
| What time in the day are you most sleepy? (e.g. 4pm – 6pm) |  |

| Participant no: |
| --- |

| **SLEEP AND ACTIVITY DIARY**  (Please complete in the **MORNING**) | **Day 1** | **Day 2** | **Day 3** | **Day 4** |
| --- | --- | --- | --- | --- |
|  | DDMM | DDMM | DDMM | DDMM |
| What time did you get into bed last night? | HHMM | HHMM | HHMM | HHMM |
| What time did you turn off the lights last night? | HHMM | HHMM | HHMM | HHMM |
| What time did you try to go to sleep? | HHMM | HHMM | HHMM | HHMM |
| How many minutes did it take for you to fall asleep last night? | min | min | min | min |
| How many times did you wake up last night? |  |  |  |  |
| How many minutes were you awake last night? | min | min | min | min |
| What time did you wake up this morning? | HHMM | HHMM | HHMM | HHMM |
| What time did you get out of bed this morning? (HH:MM) | HHMM | HHMM | HHMM | HHMM |
| How would you rate your sleep last night? (Please circle) | Terrible | Terrible | Terrible | Terrible |
|  | Poor | Poor | Poor | Poor |
|  | Good | Good | Good | Good |
|  | Excellent | Excellent | Excellent | Excellent |
