# Supplementary material for: Circadian rest-activity rhythm disorders in advanced cancer: assessment, diagnosis and clinical correlates
Source: BMJ Support Palliat Care. 2025 Oct 2;16(1):e005410. doi: 10.1136/spcare-2025-005410 (PMC12772542; doi:10.1136/spcare-2025-005410)
Supplement: online supplemental file 2 [file spcare-16-1-s002.docx]

**Appendix 2. Participants baseline characteristics (n = 65).**

| **Demographics** | **Age** | Mean: 62 years old (SD 11.61, range 38-86) |
| --- | --- | --- |
|  | **Sex** | Female (n = 35, 54%), Male (n = 30, 46%) |
|  | **Ethnicity** | White Irish (n = 60, 92.3%)  White British (n = 2, 3.1%)  White Romanian (n = 1, 1.5%)  White Other (n = 1, 1.5%)  Asian (n = 1, 1.5%) |
| **Medical characteristics** | **Cancer diagnosis** | Gastrointestinal (n = 18, 27.7%)  Breast (n = 14, 21.5%)  Lung (n = 13, 20%)  Prostate (n = 9, 13.8%)  Renal (n = 4, 6.2%)  Gynaecological (n = 4, 6.2%)  Skin (n = 2, 3.1%)  Synchronous breast/thyroid (n = 1, 1.5%) |
|  | **European Cooperative**  **Oncology Group (ECOG) performance status** | ECOG 0 (n = 12, 18%)  ECOG 1 (n = 40, 62%)  ECOG 2 (n = 11, 17%)  ECOG 3 (n = 2, 3%) |
|  | **Primary tumour in situ** | Yes (n = 33, 51%), No (n = 32, 49%) |
|  | **Location of metastatic sites** | Organ involvement (n = 44, 68%)  Bone involvement (n = 29, 45%)  Lymph node involvement (n = 30, 46%)  Cerebral involvement (n = 7, 11%)  Adrenal involvement (n = 3, 5%) |
|  | **Radiological disease status** | Improving (n = 20, 31%)  Progressive (n = 20, 31%)  Stable (n = 17, 26%)  Not applicable (n = 8, 12%) |
|  | **Biochemical disease status** | Not applicable (n = 31, 48%)  Stable (n = 14, 22%)  Progressive (n = 12, 18%)  Improving (n = 8, 12%) |
|  | **Current anticancer therapy** | Receiving anticancer therapies (n = 54, 83%)  Hormonal therapy (n = 11, 17%)  Hormonal therapy & protein kinase inhibitor (n = 5, 8%)  Hormonal therapy & monoclonal antibody (n = 1, 2%)  Chemotherapy & monoclonal antibody (n = 10, 15%)  Chemotherapy alone (n = 9, 14%)  Chemotherapy & radiotherapy (n = 2, 3%)  Chemotherapy & hormonal therapy (n = 1, 2%)  Monoclonal antibody alone (n = 8, 12%)  Kinase inhibitor alone (n = 7, 11%)  Off anticancer therapies (n = 11, 17%) |
|  | **Time since last anticancer therapy** | Continuous (n = 20, 31%)  <1 week (n = 19, 29%)  1-2 weeks (n = 9, 14%)  2-4 weeks (n = 6, 9%)  Off treatment (n = 10, 15%)  Unknown (n = 1, 2%) |
|  | **Previous treatments** | Chemotherapy (n = 45, 69%)  Radiotherapy (n = 41, 63%)  Surgery (n = 34, 52%)  Immunotherapy (n = 30, 46%)  Hormonal therapy (n = 24, 37%)  Targeted therapy (n = 19, 29%) |
|  | **Perceived weight loss in the last month** | Yes (n = 22, 34%), No (n = 43, 66%) |
| **Socioeconomic and lifestyle** | **Employment status** | Retired (n = 26, 40%)  Unemployed (n = 25, 38%)  Employed (n = 14, 22%) |
|  | **Educational attainment** | Primary school (<12 years old) (n = 2, 3%)  Junior cycle (12-15 years old) (n = 17, 26%)  Senior cycle (16-18 years old) (n = 11, 17%)  Post-senior cycle (n = 12, 19%)  Apprenticeship (n = 2, 3%)  Third degree level (n = 21, 32%) |
|  | **Caring role** | Yes (n = 13, 20%), No (n = 52, 80%) |
|  | **Living arrangements** | Alone (n = 12, 18%), With others (n = 53, 82%) |
|  | **Young children in the home** | Yes (n = 11, 17%), No (n = 54, 83%) |
|  | **Sleeping arrangements** | Own bedroom (n = 34, 52%), Shared bedroom (n = 31, 48%) |
|  | **Smoking status** | Smoker (n = 10, 15%)  Non-smoker (n = 55, 85%)  Median time of last cigarette of the day - 22:50 |
|  | **Caffeine status** | Caffeine drinker (n = 61, 94%)  Non-caffeine drinker (n = 4, 6%)  Median time of last caffeinated drink of the day -18:00 |
|  | **Alcohol status** | Alcohol drinker (n = 25, 38%)  Non-alcohol drinker (n = 40, 62%)  Median timing of last alcoholic drink of the day - 21:30 |
| **Examination** | **Body mass index (kg/m^2^)** | Underweight (< 18.5) (n = 3, 5%)  Healthy weight (18.5-24.9) (n = 20, 31%)  Overweight (25.0-30) (n = 23, 35%)  Obesity (> 30.0) (n = 19, 29%) |
|  | **Neck size (cm)** | Mean 38.18 (SD 4.07, range 30-49) |
|  | **Heart rate (beats per minute)** | Mean 80 (SD 14.42, range 48-120) |
| **Other** | **Brief Pittsburgh Sleep Quality Index (bPSQI)** | Median 5 (range 0-15) |
|  | **Epworth Sleepiness Scale (ESS)** | Median 4 (range 0-21)  Lower normal levels of daytime sleepiness (60%)  Higher normal levels of daytime sleepiness (28%)  Mild excessive levels of daytime sleepiness (8%)  Moderate excessive levels of daytime sleepiness (1%)  Severe levels of daytime sleepiness (3%) |
|  | **STOP-Bang** | High risk (n = 8, 12%)  Intermediate risk (n = 25, 39%)  Low risk (n = 32, 49%) |
|  | **Restless Leg Syndrome criteria** | Met (n = 11, 17%), Not met (n = 54, 83%) |
|  | **Chronotype (MEQ)** | Definite morning (n = 6, 9%)  Moderate morning (n = 24, 37%)  Intermediate (n = 33, 51%)  Moderate evening (n = 2, 3%) |

SD: standard deviation
